# Greater medial arterial supply revealed by 7‐Tesla quantitative magnetic resonance imaging, histology and high‐resolution computed tomography of the patellar tendon

**DOI:** 10.1002/jeo2.70668

**Published:** 2026-03-10

**Authors:** Maximilian M. Mueller, Craig E. Klinger, Sebastian Conner‐Rilk, Jerry Wang, Kevin G. Shea, Gregory S. DiFelice, Ryan Brown, Maneeza Bilal, Peter K. Sculco, Scott A. Rodeo, Daniel W. Green

**Affiliations:** ^1^ Department of Orthopaedic Surgery, Hospital for Special Surgery, NewYork‐Presbyterian Weill Medical College of Cornell University New York New York USA; ^2^ Department of Trauma Surgery Orthopaedics and Sports Traumatology, BG Klinikum Hamburg Hamburg Germany; ^3^ Medical University of Vienna Vienna Austria; ^4^ OCM—Orthopedic Surgery Munich Munich Germany; ^5^ Department of Pathology and Lab Medicine Weill Cornell Medicine New York USA; ^6^ Department of Orthopaedics Stanford University School of Medicine Stanford California USA; ^7^ Bernard and Irene Schwartz Center for Biomedical Imaging, Department of Radiology New York University Grossman School of Medicine New York New York USA; ^8^ Tissue Engineering, Repair, and Regeneration Program Hospital for Special Surgery New York New York USA

**Keywords:** arterial supply, parapatellar approach, patellar tendon, patellar tendon rupture, rupture, vascularity

## Abstract

**Purpose:**

To quantitatively assess relative arterial contributions to the patellar tendon (PT) across predefined anatomic regions with 7‐Tesla quantitative magnetic resonance imaging (7T‐qMRI), algorithm‐based histological analysis and high‐resolution computed tomography (micro‐CT) in a cadaveric model.

**Methods:**

Seven fresh‐frozen human cadaveric knee pairs (mean age 41.9 ± 15.5 years) underwent limited vascular dissection and arterial cannulation. Pre‐ and post‐contrast 7T‐qMRI, with a volumetric interpolated breath‐hold examination (VIBE) three‐dimensional T1‐weighted gradient echo pulse sequence, quantified tendonous vascularity by measuring contrast enhancement. Subsequent quantitative algorithm‐based histologic analysis with hematoxylin and eosin (H&E) staining followed, and two additional specimens underwent high‐resolution (98 μm) micro‐CT for qualitative vascular assessment.

**Results:**

In the transverse analysis, 7T‐qMRI demonstrated the highest mean relative arterial contributions in the medial region (42.4%) compared with the middle region (30.2%; *p* = 0.035) and higher, though not significant, than the lateral region (32.0%). The central PT demonstrated greater relative arterial contributions (37.5%) than the proximal (26.5%) or distal (29.3%) thirds (*p* > 0.05) in the longitudinal analysis. At the patellar enthesis, the middle third exhibited higher contributions (35.3%) than medial (28.8%) or lateral (29.6%), without significance, while the tibial tuberosity showed greater contributions along the lateral region (37.2%; *p* > 0.05). Histology confirmed significantly greater medial arterial contribution, with 8.3% higher supply than lateral (*p* = 0.018). Micro‐CT revealed a robust vascular network along the medial PT with smaller branches laterally. Distal to the inferior patellar pole, a peripatellar circular network, extending medially into the posterior PT layers, was qualitatively identified.

**Conclusion:**

7T‐qMRI and histological analyses demonstrated significantly greater arterial supply along the medial border of the PT, while micro‐CT revealed a medial and peripatellar circular vascular network extending from the medial margin and the inferior patellar pole into the posterior tendon layers. These findings identify the medial margin as the main vascular source for the PT, with implications for surgical preservation and reducing PT devascularization risk.

**Level of Evidence:**

N/A.

## Graphical abstract

7T‐qMRI and histological analyses demonstrated significantly greater arterial supply along the medial border of the patellar tendon, while micro‐CT revealed a medial and peripatellar circular vascular network.

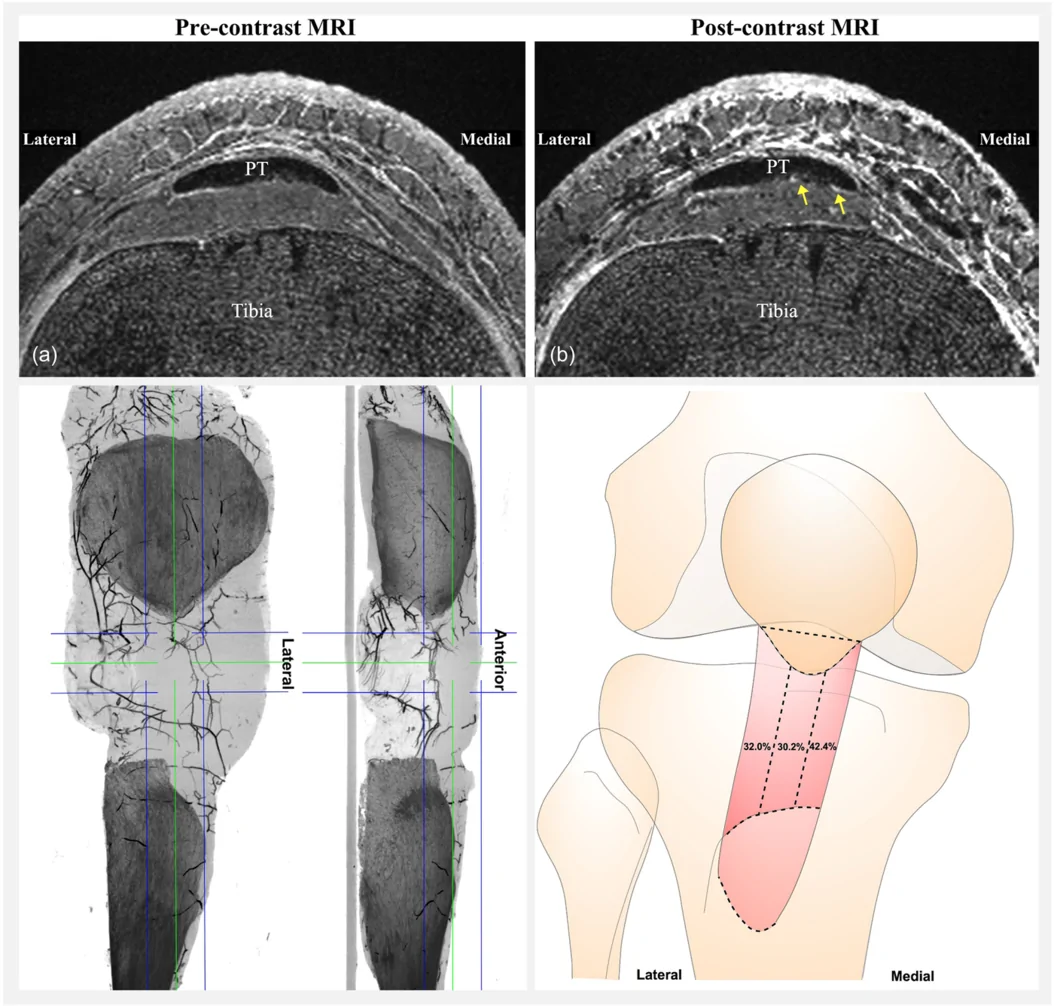

**Abbreviations**
7T‐qMRI: 7‐Tesla quantitative magnetic resonance imaging
ACL: anterior cruciate ligament
ACLR: ACL reconstruction
AVN: avascular necrosis
BPTB: bone–patellar tendon–bone
CI: confidence interval
H&E: hematoxylin and eosin
IHC: immunohistochemistry
IQR: interquartile range
Micro‐CT: high‐resolution computed tomography
PT: patellar tendon
ROI: regions of interest
SD: standard deviation
TKA: total knee arthroplasty
VIBE: volumetric interpolated breath‐hold examination

## INTRODUCTION

The patellar tendon (PT) is the distal continuation of the quadriceps tendon and an integral component of the extensor mechanism [8, 23]. Contrary to quadriceps tendon ruptures, PT ruptures are more often observed in younger, physically active populations, frequently in the setting of pre‐existing tendinopathy [4, 16]. Although acute, traumatic rupture remains the most common injury mechanism [10], iatrogenic disruption during total knee arthroplasty (TKA) has also been reported [30, 31, 35, 46].

In 1967, Scapinelli produced one of the earliest visual depictions of the peripatellar vascular network that supplies both the patella and the extensor mechanism [39]. Notably, angiographic injection technique demonstrated greater vascularity in the central portion of the tendon, which is commonly harvested for anterior cruciate ligament (ACL) reconstruction (ACLR) [34]. Extensive medial arthrotomies or lateral retinacular release may compromise the PT arterial supply, thereby significantly increasing the risk of intraoperative or postoperative tendon rupture [30, 31]. Furthermore, early PT ruptures following TKA demonstrate poor healing potential, potentially reflecting compromised vascular supply, as evidenced by high failure rates when direct tendon repair is attempted [24]. The occurrence of clinical failures after PT injury or repair indicates that the contribution of the peripheral vascular supply may have been underestimated, emphasizing the importance of its quantitative assessment. A more detailed understanding of the PT vascular supply, including quantitative comparisons of different regions within the PT, is needed to provide a critical foundation for translational research aimed at enhancing surgical approaches and mitigating treatment complications.

The study purpose was to quantitatively assess relative arterial vascular supply to the PT across predefined anatomical regions using 7‐Tesla quantitative magnetic resonance imaging (7T‐qMRI), histological analysis and high‐resolution computed tomography (micro‐CT) in a fresh‐frozen human cadaveric model. It was hypothesized that relative arterial contribution to the PT would be greater in the medial and lateral subregions compared with the central region.

## METHODS

The current study received approval from the institutional review board (2024‐1301). This study was designed as a cadaveric laboratory investigation using fresh‐frozen human specimens. Seven cadaveric knee pairs (Anatomy Gifts Registry) were obtained, sectioned from mid‐femur to mid‐tibia. Specimens with a medical history of peripheral vascular disease, metastatic malignancies, diabetes mellitus or prior knee trauma or surgery were excluded at screening by two evaluators. Causes of death included suicide (*n* = 2), drug overdose (*n* = 1), lymphoma (*n* = 1), aortic dissection (*n* = 1), cardiac arrest (*n* = 1) and undetermined (*n* = 1). All specimens were processed immediately following thawing to ensure tissue integrity. The contrast agent application prohibited micro‐CT evaluation on the same specimens as used for 7T‐qMRI and histologic analysis. Therefore, two additional knee specimens (male, 55 years; female, 58 years) were prepared for micro‐CT evaluation.

### Regions of interest (ROI)

**Figure 1:**
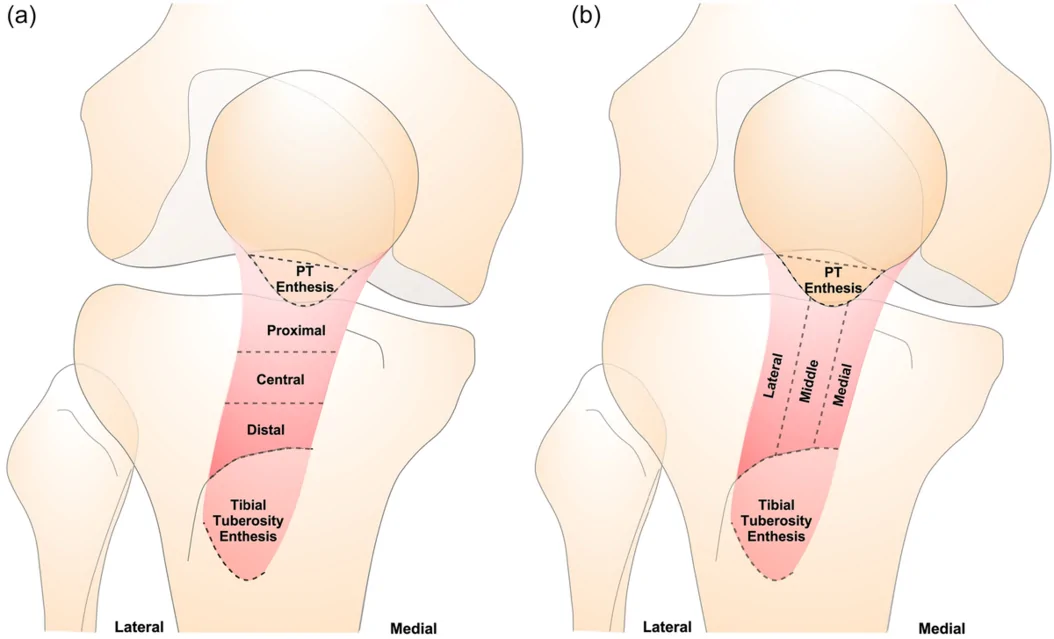
Diagram of a right‐sided patella and patellar tendon (PT) illustrating the 7‐Tesla quantitative magnetic resonance imaging (7T‐qMRI) analysis. Regions of interest (ROI) areas include (a) proximal, central and distal ROI areas, with each ROI equal in height, and (b) medial, middle and lateral ROI areas, with each ROI equal in width.

ROI were defined across all specimens by segmenting the PT into soft‐tissue and bony regions. For 7T‐qMRI, the soft‐tissue PT was manually divided into equal thirds both longitudinally (proximal, central and distal) and transversely (medial, middle and lateral). The bony ROIs were defined separately: the patellar enthesis was segmented transversely into medial, middle and lateral regions, and the tibial tuberosity was likewise divided transversely into medial, middle and lateral regions (Figure 1). For histological analysis, only the soft‐tissue PT was evaluated and segmented longitudinally into proximal, central and distal thirds, and transversely into equal medial and lateral regions.

### 7T‐qMRI analysis

**Figure 2:**
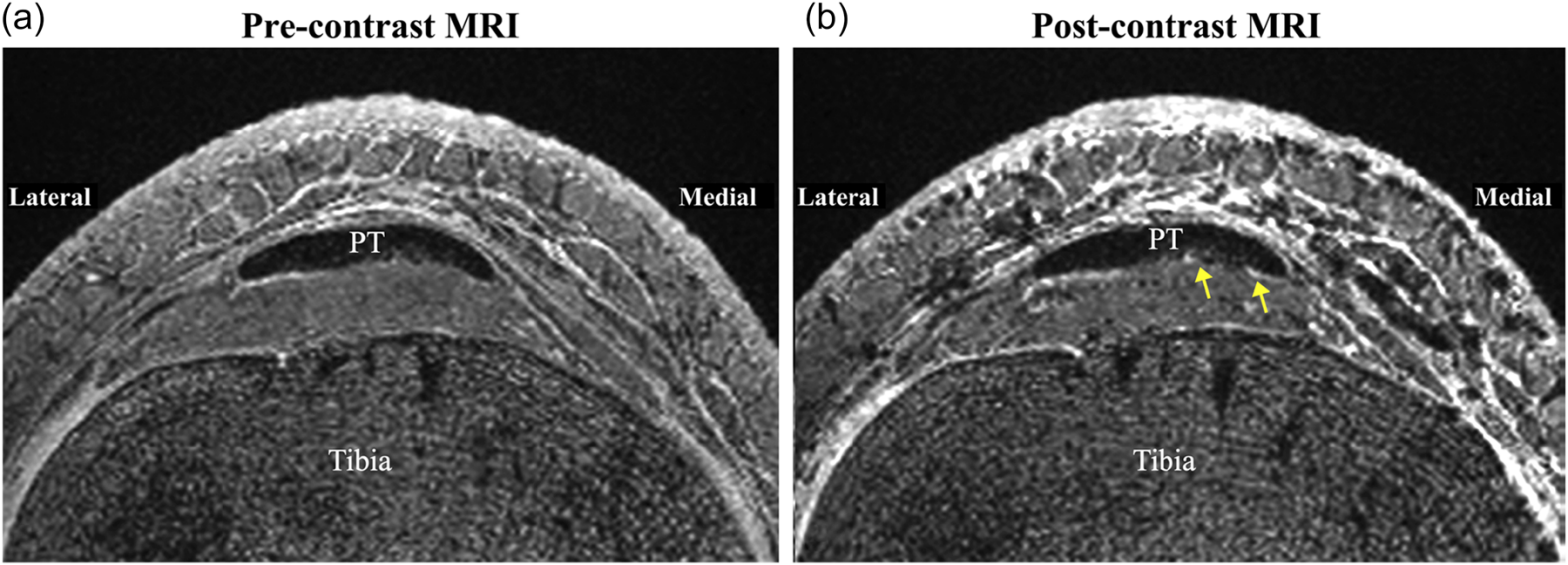
Example 7‐Tesla quantitative magnetic resonance imaging (7T‐qMRI) was acquired along axial plane. Siemens volumetric interpolated breath‐hold examination (VIBE) MRI sequence images demonstrate pre‐ (a) and post‐contrast (b) imaging of the patella tendon (PT) including increased signal at the dorsomedial border of the PT (yellow arrows).

The 7T‐qMRI analysis has previously been described and validated [25, 26, 28, 33]. Knee specimens, sectioned at the mid‐femur to mid‐tibia levels, were received at the institutional anatomy laboratory and remained intact including the skin. A limited vascular dissection was performed, starting proximally for access to the common femoral artery bifurcation to canulate the superficial femoral artery. Specimens were carefully thawed while at room‐temperature. Following supine positioning of the specimen within the knee coil, pre‐ and post‐contrast solution (61 mL, 1.5:1 saline to Gadavist; Bayer) 7T‐qMRI scans (MAGNETOM; Siemens Healthineers) were performed using a 28‐channel knee coil (Quality Electrodynamics). At the time of specimen positioning, a coiled intravenous (IV) line was pre‐filled with gadolinium solution and connected to the arterial cannula (Extension Set 001.4; Topspins Inc.). The IV lines allowed for infusion at a distance, ensuring the specimen remained unmoved during pre‐ and post‐contrast 7T‐qMRI imaging to optimize secondary quantitative image analysis. To minimize variability, all injections were completed by the same investigator using standardized syringe sizes and a consistent technique [26, 28]. Images were acquired using a product volumetric interpolated breath‐hold examination (VIBE) three‐dimensional T1‐weighted gradient echo pulse sequence with 0.45 mm isotropic resolution to quantify tendonous vascularity by measuring contrast enhancement as described in previous literature (Figure 2) [28]. MRI acquisition parameters consisted of slice thickness = 0.45 mm, echo time = 3.1 ms, repetition time = 10.9 ms, flip angle ~10°, parallel imaging acceleration factor = 4, pixel bandwidth = 550 Hz and acquisition time = 334 s, with imaging acquired along the sagittal imaging plane. The contrast solution consisted of 61 mL with a 1.5:1 ratio of saline to Gadavist (Bayer).

MRI signal intensity (SI) was measured both prior to and following contrast solution infusion.

SI was measured with software using Interactive Data Language (IDL 6.4; Exelis) on sequential slices along the sagittal image plane corresponding to each ROI. To standardize ROI measurements, MRIs were reviewed, and PT margins were assessed. The proximal and distal margins were used to divide the PT into three ROIs of equal height, and the medial and lateral margins were used to divide the PT into two ROIs of equal width. All measurements were performed at a minimum distance of 1 mm from transition zones along the medial and lateral PT border, to avoid measurement of non‐tendonous tissue. Weighted averages of SI measurements and associated histograms were generated for each pre‐ and post‐contrast MRI image and were normalized to a baseline signal derived from non‐enhancing cartilage tissue. A single measurement of signal enhancement was determined for each ROI by averaging voxel SIs across all study specimens. Finally, PT arterial contributions were quantified across all standardized ROI. For 7T‐qMRI assessment, a proximal‐to‐distal and medial‐to‐lateral analysis was performed. Further assessment of the medial‐to‐lateral arterial contributions was performed for the proximal PT due to the higher clinical frequency of PT ruptures and tendinopathy along the proximal aspect [1, 2, 32, 44].

### Algorithm‐based histological analysis

Following 7T‐MRI, tissue samples were collected for histologic processing and quantitative analysis using hematoxylin and eosin (H&E) staining. The PT was dissected and harvested from the knee specimen and segmented into six equally sized tendinous regions along the coronal plane, and both bony entheses (3 × 10 × 5 mm) were also harvested. To preserve tissue integrity, the bony entheses were not further divided. Tissue specimens were harvested with a thickness of less than 3 mm and were immediately placed in 10% neutral‐buffered formalin at room temperature for 24 h. Enthesis samples subsequently underwent decalcification for 2–3 days using ethylenediaminetetraacetic acid (EDTA). Tissue dehydration was performed through a graded ethanol series (50%–100%), followed by xylene clearing and paraffin wax infiltration. Samples were embedded within paraffin, sectioned at 5 µm thickness, and slides were baked overnight at 70° prior to staining. For each sample, one section was stained with H&E according to standard protocols. Randomly selected sections, including all ROIs, were prepared for immunohistochemistry (IHC); one with double‐staining and one negative control. IHC staining to identify and visualize blood vessels with CD31 (Leica PA0250; Leica Biosystems) was performed within 6 h of slide baking to ensure optimal antigen preservation. Slides underwent heat‐induced epitope retrieval at pH 9. Peroxide block was used to quench endogenous peroxidase activity. Secondary horseradish peroxidase (HRP)‐conjugated rabbit anti‐mouse immunoglobulin G (IgG) was applied before the Leica Bond Refine detection kit DS9800 was used for visualization (diaminobenzidine tetrahydrochloride hydrate as HRP substrate). Multiple bond wash and deionized water wash steps were added as per the default protocol. Finally, slides were counterstained with hematoxylin.

After decalcification, sectioning and H&E staining, histological slides were digitized (Aperio GT450; Leica Biosystems). A previously developed and validated algorithm (*r* = 0.95; 95% confidence interval [CI], 0.89–0.98; *p* < 0.001), optimized for vascular structure recognition in tendinous tissue, was used (https://qupath.github.io) [5, 28]. In brief, following expert consensus from three trained histology research staff on vessel identification and validation using CD31 immunohistochemical staining on serial sections, these manually annotated tiles were used to train the algorithm, which was optimized for vessel detection using a random‐forest pixel classifier. The algorithm automatically annotated each sample within each ROI and generated pixel‐based area measurements, excluding background, folded tissue regions and other artifacts (expressed as vessel area/total sample area).

### Micro‐CT

As in a previous study [28], specimens were manually infused with 500 mL of 40% barium sulphate solution (Varibar Thin Liquid; Bracco Diagnostics) mixed with 60% saline. Following infusion, the extensor mechanism was dissected and harvested, with care taken to preserve nutrient arteries. Samples then underwent micro‐CT imaging at 98 µm resolution (Inveon Multi‐Modality microPET/SPECT/CT; Siemens) for qualitative assessment of arterial anatomy across coronal, axial and sagittal image planes.

### Data analysis

SPSS (version 31; IBM) was used for statistical analysis. As part of the study design, a sample size justification was performed. Based on pilot data and prior studies [22, 28, 33], we expected a difference of ~30% between regions, with a standard deviation (SD) of 15%. With seven specimens, the study had >90% power to detect regional differences at *α* = 0.05. Under these assumptions, the minimum detectable difference between two regions was approximately 19.9% (80% power). Data were tested first for normal distribution using the Shapiro–Wilk test. Normally distributed data were expressed using mean ± SD, and in the absence of normal data distribution, median and interquartile range (IQR, 25%–75%). Additionally, 95% CI were calculated. An independent samples *t* test or ANOVA test was performed for analysis of normally distributed continuous variables and Mann–Whitney *U* or Kruskal–Wallis for nonparametric data. For all multiple comparisons, statistical significance was adjusted using the Bonferroni correction. Effect sizes for normally distributed comparisons were determined using Cohen's *d*, whereas nonparametric group comparisons were assessed using Cliff's delta to account for ordinal or skewed data. For reporting consistency, Cliff's delta values were converted to equivalent Cohen's *d* estimates. A *p* value of <0.05 was considered statistically significant.

## RESULTS

### 7T‐qMRI analysis

**Figure 3:**
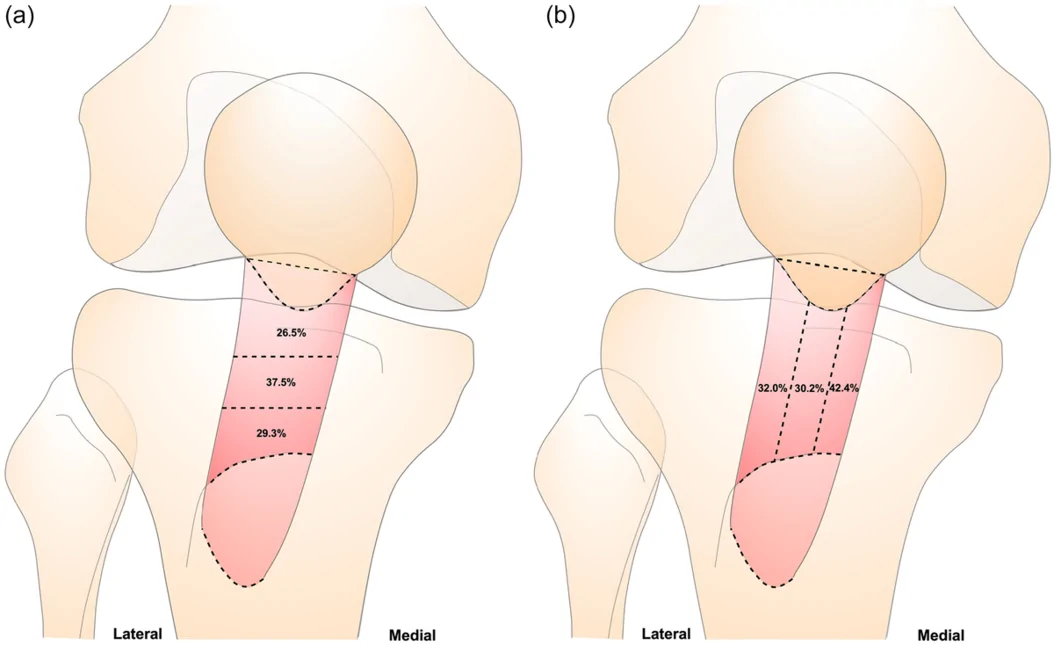
Diagram illustrating the mean relative vascular contributions to the patellar tendon of a right knee found in the 7‐Tesla quantitative magnetic resonance imaging (7T‐qMRI) proximal‐to‐distal analysis (a) and medial‐to‐lateral analysis (b).

The mean donor age of the seven included cadaveric knee pairs was 41.9 ± 15.5 years (range, 23–66 years); five donors were male, and two were female. A detailed overview of all findings of the quantitative 7T‐qMRI analysis of arterial PT contributions is provided in Table 1 and Figure 3. In the transverse assessment, regional pairwise comparisons found a significant difference between the mean arterial contribution from the medial aspect (42.4%) versus the middle aspect (30.2%; *p* = 0.035). The lateral aspect showed a slightly higher contribution compared with the middle (32%), but this difference did not reach statistical significance (*p* > 0.99) (Table 2). In the longitudinal analysis, the central ROI demonstrated higher vascular supply (37.5%) compared with the proximal (26.5%) and distal (29.3%) thirds of the PT (all *p* > 0.05). Analysis of bony entheses revealed the majority of vascularity in the middle portion of the patellar enthesis (35.3%), while the tibial tuberosity showed the highest contributions along the medial region (33.7%), although regional differences did not reach statistical significance (*p* > 0.05). The proximal PT was further assessed for medial‐to‐lateral vascular distribution and the medial aspect was found to have highest arterial contributions (43.1%, 95% CI 26.8%–86.3%), followed by central (36.3%, 95% CI 9.7%–46.0%), and with lateral the least (14.2%, 95% CI −0.9% to 32.3%). For the proximal PT, the medial arterial contribution was significantly higher than lateral (*p* = 0.032, Cohen's *d* = 1.41), while other regional differences were not significant (medial vs. central: *d* = 0.930, *p* = 0.329; lateral vs. central: *d* = −0.560, *p* > 0.999).

**Table 1:** 7T‐qMRI analysis of relative arterial contributions to the patellar tendon.

	Arterial contribution	95% CI
Patellar enthesis		
Medial	28.8% (17.9%–34.1%)	9.4%–47.4%
Middle	35.3% (33.4%–48.8%)	16.1%–70.9%
Lateral	29.6% (19.8%–37.5%)	11.8%–44.5%
Tibial tuberosity enthesis		
Medial	33.7% (25.5%–55.6%)	16.7%–56.4%
Middle	20.7% (17.5%–24.0%)	9.5%–43.1%
Lateral	31.1% (25.7%–52.3%)	14.7%–59.7%
Patellar tendon region from medial to lateral		
Medial	42.4% (36.0%–44.1%)	25.6%–69.6%
Middle	30.2% (13.1%–34.0%)	8.6%–37.9%
Lateral	32.0% (21.1%–35.1%)	12.6%–45.7%
Patellar tendon from proximal to distal		
Proximal	26.5% (11.8%–41.7%)	6.4%–49.3%
Central	37.5% (18.9%–56.0%)	10.5%–70.1%
Distal	29.3% (27.1%–42.2%)	18.0%–45.7%

*Note*: Data are presented as median (IQR).

Abbreviations: CI, confidence interval; IQR, interquartile range.

**Table 2:** 7T‐qMRI pairwise comparisons of regional arterial contributions.

	Effect size	*p* value
Patellar enthesis		
Medial–middle	−0.096	0.524
Lateral–medial	−0.192	>0.999
Lateral–middle	−0.440	0.658
Tibial tuberosity enthesis		
Medial–middle	0.560	0.735
Lateral–medial	−0.040	>0.999
Lateral–middle	0.850	>0.589
Patellar tendon region from medial to lateral		
Medial–middle	1.48	**0.035**
Lateral–medial	−1.00	0.183
Lateral–middle	0.143	>0.999
Patellar tendon from proximal to distal		
Proximal–central	−0.480	0.615
Distal–proximal	0.330	0.950
Distal–central	−0.220	0.746

*Note*: Kruskal–Wallis test, significance adjusted using the Bonferroni correction. Bold values denote statistical significance at *p* < 0.05.

Abbreviation: 7T‐qMRI, 7‐Tesla quantitative magnetic resonance imaging.

### Algorithm‐based histological analysis

Algorithm‐based histological quantitative analysis aligned with the 7T‐qMRI findings, demonstrating a 8.3% higher medial contribution compared with lateral (Cohen's *d* = 1.37; *p* = 0.018) (Tables 3, 4 and 4). The central PT showed higher arterial contributions (39.3%) compared to the proximal (31.9%) and distal (28.9%); however, these differences did not reach statistical significance (all *p* > 0.05).

**Table 3:** Histologic analysis of arterial contributions to the patellar tendon.

	Arterial contribution	95% CI
Patellar tendon region from proximal to distal		
Proximal	31.9% (25.6%–39.9%)	18.9%–44.8%
Central	39.3% (30.6%–42.4%)	28.4%–46.6%
Distal	28.9% (17.7%–41.5%)	17.9%–43.5%
Patellar tendon from medial to lateral		
Medial	56.1% (51.9%–63.1%)	48.5%–66.6%
Lateral	47.8% (41.4%–48.1%)	33.4%–53.1%

*Note*: Data are presented as median (IQR).

Abbreviations: CI, confidence interval; IQR, interquartile range.

**Table 4:** Histology pairwise comparisons of regional arterial contributions.

	Effect size	*p* value
Patellar tendon region from proximal to distal		
Proximal–central	−0.180	>0.999^a^
Proximal–distal	0.110	>0.999^a^
Central–distal	0.330	>0.843^a^
Patellar tendon region from medial to lateral		
Lateral–medial	−1.37	**0.018** b

*Note*: Bold values denote statistical significance at *p* < 0.05.

^a^ Kruskal–Wallis test, significance adjusted using Bonferroni correction.

^b^ Mann–Whitney *U* test.

### Micro‐CT

**Figure 4:**
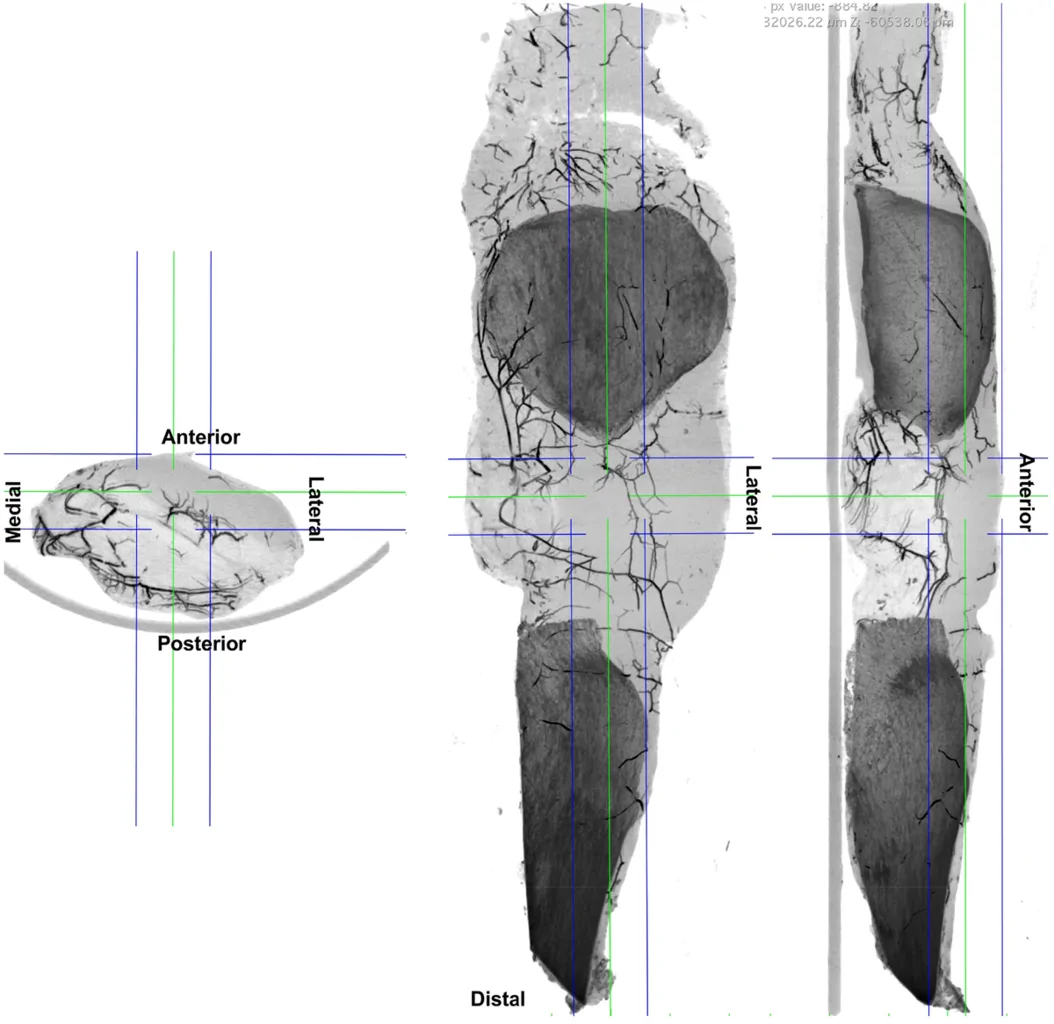
High‐resolution computed tomography (micro‐CT) multiplanar (from left: axial, coronal, sagittal) reconstruction images at 98 μm resolution in maximum intensity projection (MIP) mode demonstrating patellar tendon vascularity. A substantial network of nutrient branches was observed along the medial and posterior aspect of the patellar tendon and inferior to the patella pole.

Micro‐CT demonstrated a substantial network of nutrient branches along the medial PT and distal to the inferior pole of the patella (Figure 4). Additionally, smaller branches were observed laterally. On coronal views, a prominent circular peripatellar vascular network extending medially around the patella was visualized. Axial views revealed that these nutrient vessels originated medially and coursed predominantly into the posterior PT layers.

## DISCUSSION

The main finding of the present study was that the medial region of the PT demonstrated significantly greater vascular supply than the middle region, and that the middle region exhibited slightly lower contributions than the lateral region. In addition, micro‐CT revealed a peripatellar circular network, extending medially, particularly into the posterior PT layers. These findings highlight the importance of meticulous dissection along the medial and lateral borders of the PT to prevent iatrogenic devascularization and the associated risk of PT rupture.

Knowledge of the PT vascular supply is particularly relevant in four clinical scenarios: (1) predicting healing potential following tendon‐to‐tendon or tendon‐to‐bone repair in acute PT ruptures; (2) pathophysiology of PT tendinopathy; (3) minimizing iatrogenic PT devascularization during open surgical approaches including development of patella avascular necrosis (AVN), especially in TKA and (4) understanding donor‐site morbidity after bone–patellar tendon–bone (BPTB) graft harvest for ACLR.

While >80% of PT ruptures occur at the patella origin, injuries in the midsubstance (14.7%) or tibial tuberosity (3.2%) are rare [32]. A previous cadaveric study described a pronounced peripatellar vascular network at the inferior pole of the patella [22], suggesting high healing potential following direct tendon‐to‐bone repair. Page et al. demonstrated preserved arterial contributions following Krackow suture refixation of proximal ruptures of the PT [33]. This observation was qualitatively confirmed by the micro‐CT findings of the present study. Notably, beyond the pronounced vascularity along the medial aspect of the PT, 7T‐qMRI and histology evaluation demonstrated higher arterial contributions in the central PT, which aligns with qualitative observations, describing a retropatellar arch, which mainly supplies the middle‐third of the tendon [34, 41]. Because of the limited tendon thickness, sagittal subregions could not be distinguished in this study; however, micro‐CT suggested that the deep‐proximal portion contains a dense network of nutrient branches. These findings suggest sufficient vascularity to the central PT for midsubstance rupture healing, although surgical treatment may be technically challenging [50].

Importantly, this study found high vascularity both proximally and on the deep aspect of the tendon, areas which are commonly affected in the case of tendinopathy [1, 2, 44]. These findings may translate clinically, demonstrating that the presence of vascularity per se does not prevent pathologic changes, including the development of tendinopathy [38, 43]. Instead, PT tendinopathy is thought to develop as a result of the gradual accumulation of micro‐damage within the extracellular matrix, in a transitional zone between two mechanically dissimilar tissues, occurring in a region with inherently poor intrinsic healing capacity [6, 21, 38]. Histopathological studies describe these changes as ‘angiofibroblastic hyperplasia’, characterized by tenocyte hypercellularity and neovascularization [40, 43, 45]. Notably, however, the increased vascularity observed in affected areas appears to be ineffective in promoting tissue repair or remodelling, suggesting a maladaptive rather than regenerative vascular response [38, 43].

In open knee surgery, a medial parapatellar approach is commonly employed, which may compromise the dominant medial arterial supply to both the patella and PT [47]. This may lead to AVN of the Patella particularly at the proximal pole [9] or may lead to wound healing complications with potentially devastating consequences following TKA [29]. AVN of the patella, including cases of subsequent patellar fracture, has been attributed to excessive resection during TKA, which may compromise the patellar blood supply [49]. The current study confirms predominant medial vascularity, underlining the importance of minimizing dissection, particularly in the distal tendon region [3]. Therefore, extensor mechanism sparing approaches compared to parapatellar approaches, which preserve more of the medial soft tissue sleeve, may be beneficial [15, 17]. A lateral approach may better preserve the medial patellar blood supply; clinically, however, complication rates following TKA appear to be similar between lateral and medial approaches [13, 37]. Importantly, in several studies, lateral release in TKA has been associated with an increased risk of patella devascularization, patellar fracture and PT rupture, suggesting that the lateral side may also be vulnerable to devascularization [30, 31, 36, 42]. Accordingly, Lynch et al. recommended limiting the extent of lateral release whenever possible [27]. Furthermore, micro‐CT revealed arterial contributions arising through the Hoffa fat pad, emphasizing the importance of its preservation during surgery to avoid PT devascularization [3, 23, 33, 48].

BPTB autograft harvesting remains a common technique, particularly for ACLR in high‐demand athletes and revision cases [12]. Although PT rupture following ACLR is rare [7], donor‐site morbidity remains a concern. While donor‐site morbidity is multifactorial [18], Jones et al. reported a 31% decrease in patella vascularity following BPTB graft harvest, which likely extends to the PT [19]. Given the findings of this study, that most vascular supply originates medially and laterally, residual healing potential after graft removal remains high. Using light microscopy, Hadjicosta et al. found a mean density of 1.4 vessels/mm^2^ without regional distinction in PT grafts [14]. Furthermore, Kartus et al. found increased vascularity in central and peripheral PT regions two years after harvest, consistent with adaptive revascularization [20]. Similarly, in a canine study, higher vascularity was described in the first weeks following longitudinal incisions of the PT as a potential treatment modality for PT tendinopathy [11].

This study has limitations. First, the limited availability of cadaveric study specimens may have limited statistical power to detect significant differences between subregions and introduce selection bias in the micro‐CT analysis. Second, in the transverse histologic analysis, only two regions could be reliably distinguished, in contrast to the three regions assessed on 7T‐qMRI, which limits direct comparability between modalities. Third, the use of a cadaveric model prevents the assessment of potential tendon healing and revascularization following injury and limits conclusions regarding clinical implications. Fourth, manual contrast infusion used in this study introduces variability in perfusion pressures.

## CONCLUSION

7T‐qMRI and histological analyses demonstrated significantly greater arterial supply along the medial border of the PT, while micro‐CT revealed a medial and peripatellar circular vascular network extending from the medial margin and the inferior patellar pole into the posterior tendon layers. These findings identify the medial margin as the main vascular source for the PT, with implications for surgical preservation and reducing PT devascularization risk.

## AUTHOR CONTRIBUTIONS

All authors contributed substantially to the conceptualization and methodology of the study. Maximilian M. Mueller, Craig E. Klinger, Sebastian Conner‐Rilk, Jerry Wang, Ryan Brown and Maneeza Bilal performed the investigation. Maximilian M. Mueller and Craig E. Klinger carried out the formal analysis and writing of the original draft. Sebastian Conner‐Rilk, Jerry Wang, Ryan Brown and Maneeza Bilal revised and edited the manuscript. Kevin G. Shea, Gregory S. DiFelice, Peter K. Sculco and Scott A. Rodeo supervised, provided resources and performed critical revision and editing. Daniel W. Green performed the project administration, funding acquisition and carried out review and editing of the draft. All authors read and approved the final manuscript.

## CONFLICT OF INTEREST STATEMENT

Unrelated to this study, Kevin G. Shea serves on the Board of POSNA, holds stock options in Sarcio, Medeloop and nView, and receives research materials from Arthrex, Stryker, Evolution Surgical and Allosource. Gregory S. DiFelice receives royalties, owns stock and is a paid consultant for Zimmer Biomet. Gregory S. DiFelice receives royalties from Arthrex. Gregory S. DiFelice received stock options, provides consulting services and participates in funded research with Miach Orthopaedics. Gregory S. DiFelice receives stock options and provides consulting services for OSSIO Inc. Peter K. Sculco consults for BICMD, Inc., Enovis and Zimmer Biomet; receives royalties from Enovis and Zimmer Biomet; research support from Zimmer Biomet; serves on the advisory board of Osgenic; and holds ownership interests in BetterPT, HS2, LLC, HSS ASC Development Network, LLC, Intellijoint Surgical, Inc., Joint Effort Administrative Services Organization, LLC, Parvizi Surgical Innovation, LLC and Osgenic. Scott A. Rodeo reports consulting for Novartis, Advance Medical/Teladoc and Enovis; research support from Virginia Toulmin Foundation, OREF, Arthritis Foundation, Angiocrine Biosciences, CTSC and NIH; and stock options in Jannu Therapeutics and Overture Medical. Daniel W. Green reports roles with AONA & AO, Arthrex (consulting, royalties, speakers' bureau), OrthoPediatrics (royalties) and Wolters Kluwer (authorship). The remaining authors declare no conflict of interest.

## ETHICS STATEMENT

This study was approved by our institutional review board (2024‐1301).

## Data Availability

The data that support the findings of this study are available from the corresponding author upon reasonable request.
